# Efficacy of complex decongestive therapy for lymphedema of the lower limbs: a systematic review

**DOI:** 10.1590/1677-5449.190074

**Published:** 2020-05-29

**Authors:** Marcelo Luiz Brandão, Helen Pereira dos Santos Soares, Maria do Amparo Andrade, Ana Luísa Sabino de Campos Faria, Rayza Santos Pires

**Affiliations:** 1 Pontifícia Universidade Católica de Goiás – PUC-GO, Goiânia, GO, Brasil.; 2 Faculdade Araguaia – FA, Goiânia, GO, Brasil.; 3 Universidade Federal de Pernambuco – UFPE, Recife, PE, Brasil.

**Keywords:** linfedema, extremidade inferior, modalidades de fisioterapia

## Abstract

Lymphedema is a chronic condition that negatively affects function and quality of life. There is currently no definitive treatment. However, some options have been proposed to mitigate its consequences. Complex Decongestive Therapy (CDT) stands out as one of the main treatment methods of choice. This systematic review aimed to evaluate the effectiveness of this technique for treating lower extremity lymphedema. The results revealed that CDT was effective in reducing the volume of affected limbs. However, some questions have not yet been answered, such as: How long do patients benefit from using CDT? and How to maintain the gains obtained? It was not possible to perform a meta-analysis because of heterogeneity, unsatisfactory methodological quality of the available studies, and the lack of a gold-standard protocol for administration of the technique. Further studies are needed to advance lymphedema research and therapy.

## INTRODUCTION

Lymphedema is a build-up of water, salt, electrolytes, high molecular weight proteins, and other compounds in the interstitial compartment, because of deficient lymphatic drainage.^1^^-^4 It can be caused by congenital abnormalities or it can be an acquired condition.^1^^,^^5^

The clinical course of lymphedema can involve increased risk of infections, reduced amplitude of movement, changes to sensitivity, and reduced self-esteem.^6^^-^11 If left untreated, it can impact negatively on people’s quality of life, causing physical sequelae (overload of joints, and trophic skin ulcers), psychological conditions, and social problems, primarily when the lower limbs are involved, and placing a considerable financial burden on health and social security systems.^12^

According to the International Lymphology Society, diagnosis of lymphedema is based on clinical history and quantification (volumetry and measurements of the circumference of the limb),^2^^,^^3^^,^^13^^,^^14^ which can then be used for staging.^15^ Treatment of patients is still a challenge, since there is a lack of systematic reviews in the literature designed to determine the best treatment for reducing lymphedema of the lower limbs.^16^ Recent publications on the subject have been subject to criticism because of a lack of methodological rigor, standardized protocols, and controlled studies capable of comparing available treatments, and because of a predominance of studies focused on treatment of lymphedema of the upper limbs.^12^

In this scenario, complex decongestive therapy (CDT) is one of the most important treatment methods of choice for patients with this clinical condition, although more consistent studies are needed (such as meta-analyses) and there is also a need for protocols and adaptations that make it easier to utilize.^4^^,^^16^ There are two phases of CDT: treatment and maintenance. The first phase consists of caring for the skin, manual lymph drainage, kinesiotherapy, and bandaging the limb. Drainage can stimulate the cisterna chyli, facilitating a return of lymphatic flow.^17^^,^^18^ Kinesiotherapy is performed next, aiming to mobilize the lymph. Finally, the limb is moisturized and then compressive bandaging is conducted, aiming to create a pressure gradient in the direction of areas where more lymph absorption occurs. The second phase maintains care for the skin, physical exercises, and external compression, using bandages with varying degrees of elasticity.^15^

Against this background, the objective of this study was to assess clinical trials that used CDT specifically to treat lymphedema of the lower limbs and to analyze its efficacy by conducting a systematic review.

## METHODOLOGY

A review was conducted of articles describing systematically selected studies with clinical trial designs that used CDT. The search strategy was implemented on the following databases: Web of Science, Scientific Electronic Library Online (SciELO), MEDLINE, via PubMed, Latin American and Caribbean Health Science Literature (LILACS), OVID Technologies, Inc., and Cochrane Library. The keywords “lymphedema”, “lower extremity”, and “physical therapy modalities” were used with the Boolean operator “and”, in the following combinations: “lower extremity and lymphedema”, “lower extremity and physical therapy modalities”, “lymphedema and physical therapy modalities”, and “lower extremity and lymphedema and physical therapy modalities”. All of these combinations were used on all of the databases, searching for publications in English or Portuguese.

The review only included clinical trials, with no publication date limits, that had a group in which CDT was the primary intervention and with a control group given other treatments (care guidance, lectures providing health information, and CDT combined with other techniques). Study groups should comprise patients of both sexes, aged over 18 years, with lymphedema lasting at least 3 months in the lower limbs (unilateral or bilateral), which could have primary or secondary causes.

Two independent reviewers conducted the search and extraction and analysis of the data. Articles were selected after reading titles and abstracts and then those that did not meet the inclusion criteria were excluded. Disagreements between reviewers were resolved during consensus meetings, in the presence of a third evaluator.

Article selection was conducted using a form covering the following information: number of participants in each group (intervention and control); details of the protocols for each group; duration of CDT administration; methods used to asses limb volume; results of the primary outcome (percentage volume reduction); and number of losses to follow-up.

Qualitative analysis of data was based on the Risk of Bias tool available in the RevMan 5.3 program from Cochrane.^19^ The tool covers six criteria (randomization sequence, allocation concealment, blinding of participants, researchers, and examiners, losses from samples, and selective description of results), enabling evaluation of the methodology and quality of clinical trials and judgment of their influence on the results reported.

## RESULTS

**Figure 1:**
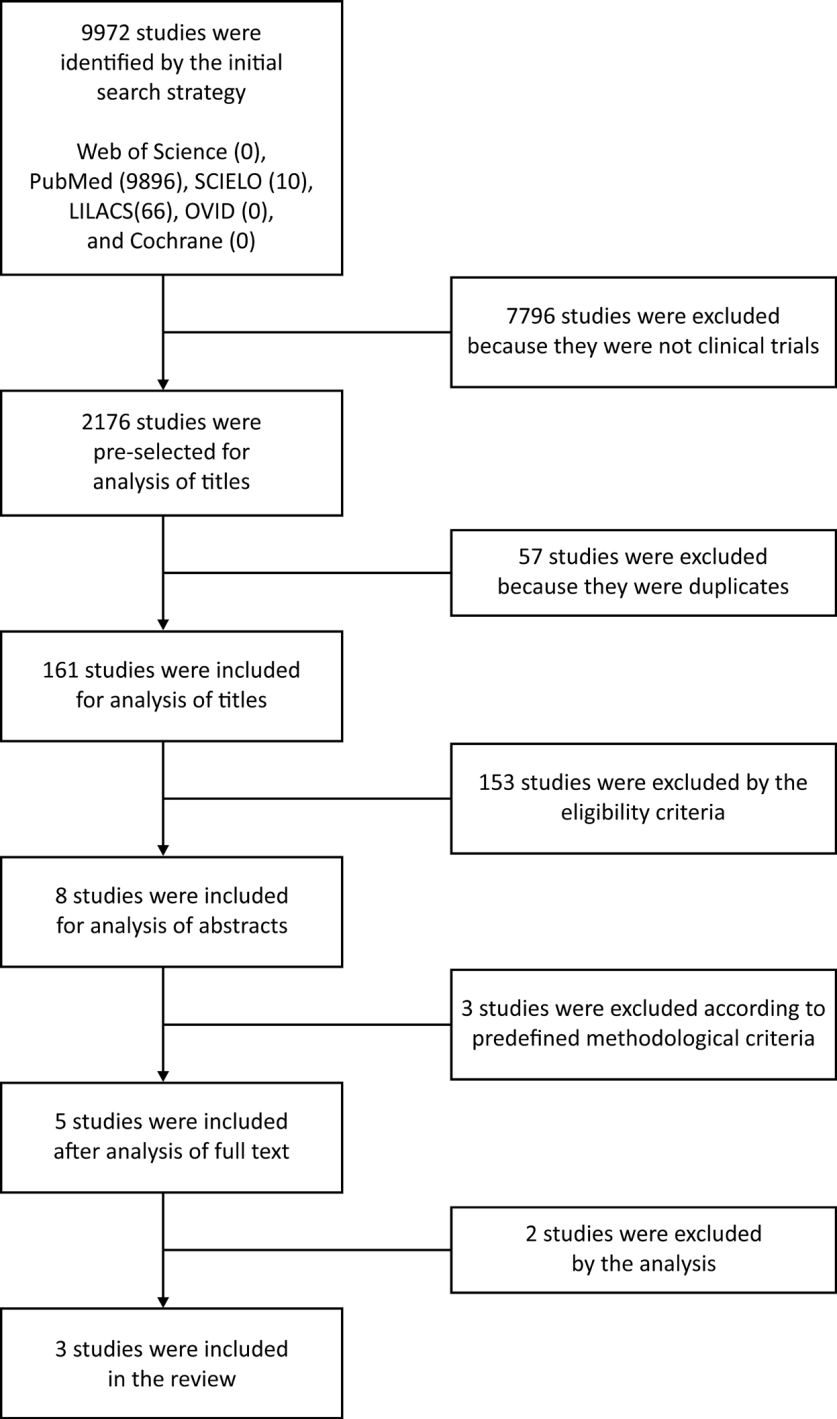
Flow diagram illustrating selection of articles.

The initial searches returned a total of 9,972 references in the literature. After selecting clinical trials, a total of 2,176 articles remained, 57 of which were duplicates. Thus, a total of 2,119 unique references were identified on the electronic databases searched. The process of selecting articles by title reduced the number to 161 studies potentially of interest. The abstracts of these 161 articles were read, identifying eight studies that met the selection criteria. After reading the full texts of these articles, with rigorous application of the inclusion protocol, just five clinical trials were found to meet the eligibility criteria. However, two articles were excluded because they did not exclusively focus on CDT for lymphedema. The search strategy is illustrated in Figure 1.

The three clinical trials selected were analyzed in two stages. First, the form was completed with details of the samples, the clinical protocols employed, and the outcomes of each article, as shown in Table 1.^16^^,^^20^^,^^21^ It was clear that the method used to assess lymphedema (volumetry) is the only parameter that is similar across all three studies.

**Table 1:** Studies included in the systematic review.

**Study**	**N intervention**	**N control**	**Details of study protocol**	**Duration of CDT administration**	**Method for assessing lymphedema in the lower limbs**	**Results (reduction of volume)**	**Losses to follow-up**
Soares et al.^16^	15	12	IG: CDT (2 x/week) CG: informative lecture	10 weeks	Volumetry + circumference measurements + QoL questionnaire	Lymphedema was reduced in the intervention group only	3
Casley-Smith et al.^20^	356	272	IG: CDT (5-6 x/week) CG: CDT + OBP or CDT + TBP or CDT + OBP + TBP	4 weeks	Volumetry	Lymphedema was reduced in both groups. However, the control groups had more intense reductions and better maintenance of results	Not reported
Tacani et al.^21^	G1: 4	G2: 3	G1: MLD + EC (1 x/week)	12 weeks	circumference measurements + volumetry before and after interventions	Lymphedema was reduced in both groups	3
			G2: CDT + ICB (2 x/week)				

N: number of patients; OBP: oral benzopyrone; TBP: topical benzopyrone; EC: elastic compression; MLD: manual lymph drainage; ICB: inelastic compression bandaging; G1: group 1; G2: group 2; CG: control group; IG: intervention group; QoL: quality of life; CDT: complex decongestive therapy.

**Figure 2:**
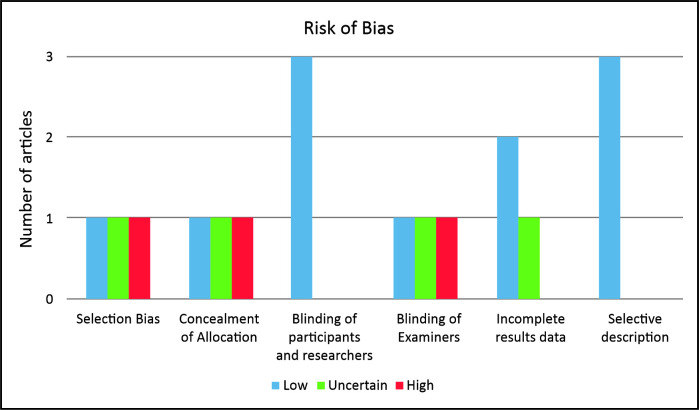
Assessment of risk of bias in articles, according to the variables covered by the Risk of Bias tool.

In the second stage of analysis, the Risk of Bias tool was applied, the results of which are illustrated in Figure 2. It was observed that one article did not describe the methods used to select participants, constituting a high risk of selection bias. Concealment of allocation to control and intervention groups was only described in one of the studies. None of the three studies blinded participants and researchers, since the type of intervention meant that a placebo treatment was no feasible, constituting a low risk of bias.

Blinding of examiners was only described in one study. The other clinical trials did not provide this information or did not conduct blinding. Only one of the clinical trials did not report losses to follow-up of participants or of limbs affected by lymphedema. As such, the qualitative analysis confirmed the heterogeneous nature of the articles and the impossibility of conducting a meta-analysis.

## DISCUSSION

Irrespective of the cause of lymphedema, it appears to be influenced by use of CDT or application of external pressure. The most relevant question is not whether or not lymphedema reduction occurs, since the studies confirm the technique’s efficacy, primarily from a clinical point of view. Rather, it is necessary to determine for how long the patients benefit from CDT and how to maintain the improvements achieved.

The study by Casley-Smith et al.^20^ is the only one in the review to describe long-term follow-up of patients after the end of the course of treatment with the technique. These researchers followed some of the participants with lymphedema for a 12-month period and were able to confirm that the benefit of CDT in terms of reduction of limb volume was sustained. Notwithstanding, patients who continued taking benzopyrones had greater reductions and more prolonged maintenance of the effects achieved during treatment.

The methodology used in the clinical trial just cited was limited for assessing CDT exclusively for the lower limbs, because it analyzed patients with lymphedema in both limbs and did not make any distinction with regard to their allocation to study groups. Moreover, the study did not describe the criterion applied to select patients who underwent a further course of CDT for 4 more weeks from those who were only reassessed after 1 year.

Tacani et al.^21^ conducted post-therapy follow-up, but only for 3 months. One of the strong points of this study was that it conducted four physiotherapy assessments (before and during CDT and during and after the maintenance period), thereby providing an illustration of the efficacy of the treatment and determination of the reductions in volume at each data collection point. However, there was an allocation bias, because the researchers distributed participants according to their lymphedema stage, which could have affected the outcome.

Compared with other results, this same study reported slightly lower percentage volume reductions. It is believed that this is related to the lower frequency of CDT sessions administered to the group and to the fact that lymphedema was at initial stages. The stand-out factor of this trial is that it was the only study in which all patients had bilateral lower limb lymphedema.

Soares et al.^16^ had a unique approach to assessment of the results. They administered a quality of life questionnaire (WHOQOL-bref)^22^ validated by the World Health Organization to both groups and concluded that the association with CDT was beneficial to the physical domain of quality of life. However, they did not observe significant differences in the results for functionality according to the timed up and go test, showing a major contradiction, in that there were no statistically favorable results, but the patients and researchers reported improvements, which was also observed in the other articles.

There is a discrepancy between the clinical improvement reported by the patients and researchers and the statistical results reported. It is believed that this is because the outcome concentrates on volumetric reduction of lymphedema, whereas patients value other parameters, such as functionality, mobility, and lower rates of complications; items that are not assessed by the majority of researchers.

One positive point in relation to assessment of the results is that all studies used volumetry, which is considered the gold standard. However, the limitations are related to differences between protocols, lack of control groups for comparison of results, and different statistical methods used to analyze the volumetric reduction. In conjunction, these factors made it impossible to determine the efficacy of CDT on its own and prevented meta-analysis.

The qualitative analysis found that none of the studies reviewed was blind. The type of intervention employed prevents blinding of patients and researchers. However, in order to minimize biases, examiners could have been blinded, but this was only specified in one of the studies.

Randomization and concealment of allocation of participants were not described in two of the studies, increasing the risk of biases. Another factor not described in one of the studies was losses to follow-up, which is a variable that could affect the outcome, since it is capable of introducing bias in the estimation of the effect of CDT. Authors mentioned losses of participants to follow-up, primarily because of erysipelas crises and of difficulty attending the sessions due to health conditions or financial problems.^15^^,^^16^^,^^20^^,^^21^ Certain measures were proposed by the authors, such as development of low-cost materials and encouraging self-care of the limb with lymphedema, but they were only tested in one article.^15^^,^^16^^,^^21^

If these clinical trials had been standardized in methodological and analytical terms, they could have been useful for answering more questions related to the effects of treatment, such as the impact of CDT on health conditions and the possible physical and psychosocial benefits for the people treated.

## CONCLUSIONS

The studies demonstrated that CDT reduced lymphedema. However, it was not possible to state the duration of its effects. The heterogeneous qualitative nature and the small number of studies selected precluded a quantitative analysis (meta-analysis). Clinical trials with greater methodological detail and follow-up of patients in the maintenance phase are needed.
