# Supplementary material for: Efficacy of complex decongestive therapy for lymphedema of the lower limbs: a systematic review
Source: J Vasc Bras. 2020 May 29;19:e20190074. doi: 10.1590/1677-5449.190074 (PMC8202203; doi:10.1590/1677-5449.190074)
Supplement: Translation

# Translation: Eficácia da terapia complexa descongestiva para linfedema nos membros inferiores: revisão sistemática

**Authors:** Marcelo Luiz Brandão, Helen Pereira dos Santos Soares, Maria do Amparo Andrade, Ana Luísa Sabino de Campos Faria, Rayza Santos Pires

**Affiliations:** 1 Pontifícia Universidade Católica de Goiás – PUC-GO, Goiânia, GO, Brasil.; 2 Faculdade Araguaia – FA, Goiânia, GO, Brasil.; 3 Universidade Federal de Pernambuco – UFPE, Recife, PE, Brasil.

**Keywords:** linfedema, extremidade inferior, modalidades de fisioterapia

## Abstract

O linfedema é uma condição crônica que afeta negativamente a função e a qualidade de vida. Atualmente, não existe tratamento definitivo. Todavia, algumas opções foram propostas para amenizar suas consequências. Entre elas, destaca-se a terapia complexa descongestiva (TCD), que se configura como um dos principais métodos de escolha de tratamento. Esta revisão sistemática teve por objetivo avaliar a eficácia dessa técnica no tratamento de linfedemas nos membros inferiores. Os resultados revelaram que a TCD foi eficaz na redução do volume das extremidades afetadas. No entanto, algumas questões ainda não foram elucidadas, tais como: por quanto tempo os pacientes se beneficiam com o uso da TCD? Como manter os ganhos obtidos? Diante da heterogeneidade, da insatisfatória qualidade metodológica dos trabalhos disponíveis e da inexistência de protocolo padrão para aplicação da técnica, não foi possível realizar a metanálise, o que demonstra que há muito a se avançar na investigação e na terapêutica do linfedema.

## INTRODUÇÃO

Linfedema é o acúmulo de água, sal, eletrólitos, proteínas de alto peso molecular e outros compostos dentro do compartimento intersticial devido ao déficit da drenagem linfática^1^^-^4. Pode ser decorrente de anormalidades congênitas ou adquiridas^1^^,^^5^.

Clinicamente, o linfedema pode cursar com aumento do risco de infecções, diminuição da amplitude de movimento, alterações sensitivas e comprometimento da autoestima^6^^-^11. Quando não tratado, pode interferir negativamente na qualidade de vida do indivíduo, causando sequelas físicas (sobrecarga articular e lesões tróficas da pele) e alterações psíquicas e sociais, principalmente quando acomete os membros inferiores, além de onerar o sistema de saúde assistencial e previdenciário^12^.

O diagnóstico do linfedema envolve história clínica e quantificação (volumetria e medição da circunferência do membro)^2^^,^^3^^,^^13^^,^^14^, com posterior estadiamento, de acordo com a Sociedade Internacional de Linfologia^15^. Já o tratamento dos pacientes permanece um desafio, uma vez que existe uma carência de revisões sistemáticas na literatura que busquem elucidar qual a melhor terapia para a redução do linfedema nos membros inferiores^16^. As recentes publicações acerca do tema foram alvo de críticas devido à falta de rigor metodológico, à escassez de padronização dos protocolos usados, à ausência de trabalhos controlados capazes de comparar as terapias disponíveis e à predominância de estudos concentrados no tratamento do linfedema nos membros superiores^12^.

Nesse contexto, a terapia complexa descongestiva (TCD) configura-se como um dos principais métodos de escolha de tratamento dos portadores dessa condição clínica, apesar de necessitar de estudos mais consistentes (como a metanálise) e de protocolos e adaptações que facilitem sua utilização^4^^,^^16^. A TCD é composta por duas fases: tratamento e manutenção. A primeira consiste em cuidados com a pele, drenagem linfática manual, cinesioterapia e enfaixamento do membro. A drenagem pode estimular a cisterna do quilo, facilitando a restituição do fluxo linfático^17^^,^^18^. Em seguida, é realizada a cinesioterapia, visando a mobilização da linfa. Por fim, hidrata-se o membro, iniciando o enfaixamento compressivo com bandagens, no intuito de criar uma coluna de pressão em direção às áreas em que ocorre maior absorção da linfa. A segunda fase mantém os cuidados com a pele, a aplicação de exercícios físicos e a compressão externa, com uso de faixas de diferentes níveis de elasticidade^15^.

Diante dessas considerações, o objetivo deste estudo foi avaliar ensaios clínicos que utilizaram especificamente a TCD para tratar linfedemas nos membros inferiores e analisar a sua eficácia por meio de uma revisão sistemática.

## METODOLOGIA

Foi realizada uma revisão de artigos com desenho de ensaio clínico, selecionados sistematicamente, que utilizaram a TCD. A estratégia de busca foi feita nas bases de dados Web of Science, Scientific Eletronic Library Online (SciELO) e MEDLINE por meio da PubMed, Latin American and Caribbean Health Science Literature (LILACS), OVID Technologies, Inc. e Cochrane Library. Foram usados os descritores “lymphedema”, “lower extremity” e “physical therapy modalities”, acrescentando o boleador “and”, com as seguintes combinações: “lower extremity and lymphedema”, “lower extremity and physical therapy modalities”, “lymphedema and physical therapy modalities” e “lower extremity and lymphedema and physical therapy modalities”. Todos esses cruzamentos foram utilizados nas bases de dados eleitas para busca em inglês e português.

A revisão incluiu somente ensaios clínicos, sem limite de data de publicação, que tiveram como intervenção a TCD no grupo principal e apresentaram grupo controle recebendo outros métodos de tratamento (orientações de cuidados, palestras com informações a respeito de saúde e TCD combinada com outras técnicas). Os grupos deveriam conter pacientes de ambos os sexos, com idade superior a 18 anos, com permanência do linfedema por, pelo menos, 3 meses em membros inferiores (uni ou bilateral), podendo ser de causa primária ou secundária.

Dois revisores independentes realizaram a busca, a extração e a análise dos dados. Os artigos foram selecionados com base na leitura de títulos e resumos; posteriormente, aqueles que não contemplavam os critérios de inclusão foram excluídos. As divergências entre os avaliadores foram tratadas em reuniões de consenso, com a presença de um terceiro avaliador.

A seleção dos artigos foi realizada por meio de um formulário contendo as seguintes informações: número de participantes em cada grupo (intervenção e controle); detalhamento do protocolo dos grupos; tempo de aplicação da TCD; métodos de avaliação do volume do membro; resultados do desfecho principal (redução do percentual de volume); e número de perdas no decorrer dos estudos.

A análise qualitativa dos dados foi fundamentada na ferramenta Risk of Bias, do programa RevMan, versão 5.3 da Cochrane^19^. Tal ferramenta considera seis critérios (sequência de randomização, ocultação de alocação, cegamento dos participantes, pesquisadores e avaliadores, perdas amostrais e descrição seletiva dos resultados), possibilitando avaliar a metodologia e a qualidade dos ensaios clínicos e, por fim, verificar sua influência nos resultados relatados.

## RESULTADOS

Inicialmente, foram encontradas 9.972 referências na literatura. Após a seleção dos ensaios clínicos, foram obtidos 2.176 artigos, sendo que, desses, 57 estavam em duplicidade. Assim, no total, 2.119 referências únicas foram encontradas nas bases de dados eletrônicas pesquisadas. Em seguida, realizou-se a avaliação por títulos, sendo selecionados 161 estudos de potencial interesse. Procedeu-se à leitura do resumo desses 161 artigos, com identificação de oito trabalhos capazes de preencher os critérios de seleção. Após a leitura integral desses, com rigorosa avaliação do protocolo de inclusão, somente cinco ensaios clínicos contemplaram os critérios de elegibilidade. Entretanto, dois artigos foram excluídos por não abordarem a TCD exclusivamente no linfedema. A representação esquemática do resultado da busca encontra-se na Figura 1.

**Figura 1:** Fluxograma de seleção dos artigos.

A análise dos três ensaios clínicos selecionados foi realizada em duas etapas. Primeiro, aplicou-se o formulário, em que foi possível conhecer as amostras, os protocolos clínicos utilizados e os desfechos de cada artigo, conforme exemplificado na Tabela 1 16^,^^20^^,^^21^. Foi possível constatar que o método de avaliação do linfedema (volumetria) é o único parâmetro semelhante entre os trabalhos.

**Tabela 1:** Estudos incluídos na revisão sistemática.

**Estudo**	**N intervenção**	**N controle**	**Detalhamento do protocolo**	**Tempo de aplicação da TCD**	**Método de avaliação do linfedema nos membros inferiores**	**Resultados (na redução de volume)**	**Perdas de seguimento**
Soares et al.^16^	15	12	GI: TCD (2 x/semana) GC: palestra informativa	10 semanas	Volumetria + perimetria + questionário de QV	Apenas o grupo intervenção reduziu o linfedema	3
Casley-Smith et al.^20^	356	272	GI: TCD (5-6 x/semana) GC: TCD + BPO ou TCD + BPT ou TCD + BPO + BPT	4 semanas	Volumetria	Ambos os grupos reduziram o linfedema. Porém, os grupos controle apresentaram uma redução mais intensa e melhor manutenção dos resultados	Não relata
Tacani et al.^21^	G1: 4	G2: 3	G1: DLM + CE (1 x/semana)	12 semanas	Perimetria + volumetria antes e após intervenções	Ambos os grupos reduziram o linfedema	3
			G2: TCD + ECI (2 x/semana)				

N: número de pacientes; BPO: benzopirona oral; BPT: benzopirona tópica; CE: compressão elástica; DLM: drenagem linfática manual; ECI: enfaixamento compressivo inelástico; G1: grupo 1; G2: grupo 2; GC: grupo controle; GI: grupo intervenção; QV: qualidade de vida; TCD: terapia complexa descongestiva.

Na segunda etapa, foi aplicada a ferramenta Risk of Bias e seus resultados são apresentados na Figura 2. Constatou-se que um artigo não apresentou a forma como os participantes foram selecionados, constituindo alto risco de viés de seleção. O sigilo de alocação dos grupos controle e intervenção foi descrito em apenas um estudo. Nenhum dos três estudos fez o cegamento dos participantes e pesquisadores, uma vez que o tipo de intervenção proposta inviabiliza a terapia por placebo, constituindo, assim, baixo risco de viés.

**Figura 2:** Avaliação do risco de viés dos artigos de acordo com as variáveis propostas pela ferramenta Risk of Bias.

O cegamento dos avaliadores foi descrito em apenas um estudo. Os demais ensaios clínicos não expõem tal informação ou não realizaram o cegamento. Apenas um ensaio clínico não declarou as perdas de seguimento dos participantes ou a extremidade afetada pelo linfedema. Assim, a análise qualitativa confirmou a heterogeneidade dos artigos, impossibilitando a realização de metanálise.

## DISCUSSÃO

Os linfedemas, independentemente da causa, parecem sofrer influência com o uso da TCD ou da aplicação de pressão externa. A questão mais relevante não se concentra na redução do linfedema, visto que os estudos comprovam a eficácia da técnica, principalmente do ponto de vista clínico. Torna-se necessário saber por quanto tempo os pacientes se beneficiam com o uso da TCD e como manter os ganhos obtidos.

O estudo de Casley-Smith et al.^20^ é o único da revisão que apresenta seguimento dos pacientes em longo prazo, após o fim da aplicação da técnica. Esses pesquisadores acompanharam alguns participantes com linfedema por um período de 12 meses, sendo possível comprovar a manutenção do benefício da TCD na redução do volume dos membros. Contudo, os pacientes que continuaram em uso de benzopiroides obtiveram maiores ganhos e manutenção prolongada dos efeitos adquiridos durante o tratamento.

A metodologia usada no ensaio clínico supracitado se torna limitante para avaliação da TCD exclusivamente em membros inferiores, pois analisa pacientes com linfedema em ambos os membros e não faz distinção quanto a sua alocação nos grupos. Além disso, o estudo não evidencia o critério de seleção dos pacientes que seriam novamente submetidos a TCD por mais 4 semanas daqueles que seriam reavaliados apenas após 1 ano.

Tacani et al.^21^ realizaram seguimento pós-terapia, mas por apenas 3 meses. Um dos destaques do trabalho é que ele faz quatro avaliações fisioterapêuticas (antes e durante a TCD e durante e após o período de manutenção), permitindo, assim, visualizar a eficácia do tratamento e acompanhar a redução do volume em cada período. Entretanto, existe um viés de alocação, pois os pesquisadores distribuíram os participantes de acordo com o estágio do linfedema, podendo haver interferências no desfecho.

Em comparação a outros resultados, esse mesmo estudo descreve um percentual de redução do volume um pouco menor. Acredita-se que isso esteja relacionado à menor frequência de sessões da TCD recebidas pelo grupo e ao fato de o linfedema estar no estágio inicial. O diferencial consiste neste ensaio ser o único que apresenta linfedema bilateral de membros inferiores em todos os pacientes.

Soares et al.^16^ apresentam singularidades na avaliação dos resultados. Os pesquisadores aplicaram o questionário de qualidade de vida (WHOQOL-bref)^22^, validado pela Organização Mundial da Saúde, em ambos os grupos, concluindo que a associação com a TCD é benéfica no domínio físico da qualidade de vida. Contudo, não apresentou resultados significativos em relação à funcionalidade pelo teste *timed up and go*, mostrando uma grande contradição, que é a ausência de resultados estatisticamente favoráveis, apesar do relato de melhora pelos pacientes e pesquisadores, condição também observada nos demais artigos.

Existe uma divergência entre a melhora clínica relatada por pacientes e pesquisadores em relação aos resultados estatísticos apresentados. Acredita-se que isso ocorre pelo fato de o desfecho concentrar-se na redução volumétrica do linfedema e o paciente valorizar outros parâmetros, como funcionalidade, mobilidade e menor ocorrência de complicações, itens que não são avaliados pela maioria dos pesquisadores.

Um ponto positivo em relação à avaliação dos resultados é que todos os estudos utilizaram a volumetria, que é considerada o padrão-ouro. Contudo, as limitações concentram-se nas divergências de protocolos, na falta de grupo controle para comparação dos resultados e nos diferentes métodos estatísticos na análise da redução volumétrica. Esse conjunto de fatores impossibilita o conhecimento da eficácia da TCD isoladamente, bem como a realização da metanálise.

A avaliação qualitativa mostra que nenhum dos estudos da revisão foi cego. O tipo de intervenção realizada impede o cegamento de pacientes e de pesquisadores. Entretanto, para minimizar os vieses, poderia ter ocorrido o cegamento dos avaliadores, o que somente é especificado em um estudo.

A randomização e o sigilo de alocação dos participantes não foram descritos em dois dos ensaios, aumentando o risco de vieses. Outro ponto não abordado em um dos estudos foi a perda de seguimento, variável que pode influenciar no desfecho, sendo capaz de induzir viés na estimativa do efeito da TCD. Os autores citam a perda de seguimento dos participantes devido, principalmente, ao desenvolvimento de crises de erisipela e à dificuldade de comparecer às sessões por agravos à saúde e pelas precárias condições financeiras dos pacientes^15^^,^^16^^,^^20^^,^^21^. Algumas medidas foram propostas pelos autores, como o desenvolvimento de materiais de baixo custo e o incentivo ao autocuidado com o membro com linfedema, mas somente foram testadas em um artigo^15^^,^^16^^,^^21^.

Se os ensaios clínicos apresentassem uma padronização metodológica e analítica, poderiam ser úteis para responder mais questões relativas aos efeitos do tratamento, como o impacto da TCD nas condições de saúde e possíveis benefícios físicos e psicossociais nos indivíduos tratados.

## CONCLUSÃO

Os estudos demonstraram que a TCD reduziu o linfedema. Contudo, não foi possível afirmar qual a duração dos seus efeitos. A heterogeneidade qualitativa, bem como a reduzida quantidade de trabalhos selecionados, impossibilitou a análise quantitativa (metanálise). Fazem-se necessários ensaios clínicos que apresentem detalhamento metodológico e que acompanhem os pacientes na fase de manutenção.
